# Cardinal lessons in cardiac amyloidosis: diagnostic pitfalls across three cases—a case report

**DOI:** 10.1093/ehjcr/ytag654

**Published:** 2026-09-02

**Authors:** Ghasaq Saleh, Omar Abou Ezzeddine, Fadi Adel

**Affiliations:** Department of Cardiovascular Medicine, Mayo Clinic, 200 First St. SW, Rochester, MN 55905, USA; Department of Cardiovascular Medicine, Mayo Clinic, 200 First St. SW, Rochester, MN 55905, USA; Department of Cardiovascular Medicine, Mayo Clinic, 200 First St. SW, Rochester, MN 55905, USA

**Keywords:** Case report, Cardiac amyloidosis, ATTR-CM, AL-CM

## Abstract

**Background:**

Cardiac amyloidosis (CA) is an infiltrative cardiomyopathy caused by extracellular deposition of misfolded amyloid fibrils within the myocardium, resulting in progressive ventricular wall thickening and heart failure. Transthyretin (ATTR) and immunoglobulin light-chain (AL) amyloidosis account for ∼95% of cases. Despite advances in diagnostic imaging and laboratory testing, CA remains underrecognized, often leading to misdiagnosis or delayed diagnosis. Timely and accurate diagnosis, including amyloid subtyping, is critical to guide appropriate therapy and improve clinical outcomes, as prognosis and management vary substantially among amyloid subtypes.

**Case summary:**

We present a three-case series illustrating distinct diagnostic and clinical challenges in CA, including a false-positive diagnosis of ATTR cardiomyopathy (ATTR-CM) based on planar technetium-99m pyrophosphate (PYP) scintigraphy that was subsequently ruled out by single-photon emission computed tomography (SPECT) imaging and further evaluation, a delayed diagnosis of AL amyloidosis in a patient with progressive heart failure initially attributed to non-ischaemic cardiomyopathy, and recurrent left atrial appendage thrombus despite therapeutic anticoagulation in a patient with AL amyloidosis.

**Discussion:**

These three cases demonstrate the broad spectrum of diagnostic and clinical challenges encountered in CA. The first case underscores the importance of SPECT imaging in differentiating true myocardial radiotracer uptake from blood pool activity, a common source of false-positive planar PYP studies. The second emphasizes the critical role of comprehensive monoclonal protein screening in patients with suspected amyloidosis as delayed diagnosis of AL amyloidosis may adversely affect outcomes. The third illustrates the heightened thromboembolic risk associated with CA, even in the setting of therapeutic anticoagulation, and supports consideration of transoesophageal echocardiography before cardioversion in this high-risk population.

Learning points

- Planar technetium-99m pyrophosphate (PYP) scintigraphy may yield false-positive findings, underscoring the importance of single-photon emission computed tomography in distinguishing myocardial uptake from blood pool activity.
- Initial diagnostic work-up for cardiac amyloidosis requires simultaneous evaluation for transthyretin and immunoglobulin light-chain amyloidosis using PYP scintigraphy and monoclonal protein testing.
- Cardiac amyloidosis is associated with a highly thrombogenic atrial substrate; therefore, transoesophageal echocardiography should be considered before direct current cardioversions regardless of anticoagulation status.

## Introduction

Amyloidosis is a broad group of diseases characterized by the extracellular accumulation of insoluble, misfolded proteins called amyloid fibrils in tissues. These proteins can deposit locally or systemically, as in cardiac amyloidosis (CA).^1^ Cardiac amyloidosis results from abnormal extracellular deposition of amyloid fibrils in the myocardium. There are two main forms that involve the heart, distinguished by the type of precursor protein involved: immunoglobulin light-chain (AL) amyloid, which is derived from light chains secreted by monoclonal plasma cells, and transthyretin (ATTR) amyloid, which originates from misfolded monomers or oligomers of the liver-produced protein transthyretin (TTR, also known as prealbumin).^1^ Wild-type ATTR (ATTRwt) amyloidosis, formerly known as senile systemic amyloidosis, is the most common form of CA and frequently misdiagnosed as heart failure with preserved ejection fraction.^1,2^

Cardiac amyloidosis contributes to cardiac dysfunction through multiple mechanisms.^3^ The extracellular accumulation of amyloid fibrils within the interstitial spaces increases ventricular wall thickness and stiffness, leading to impaired diastolic function. In AL amyloidosis, amyloid deposits may also involve intramyocardial arterioles, resulting in angina or, more rarely, myocardial infarction. Additionally, atrial infiltration by amyloid fibrils promotes interstitial remodelling, creating a substrate for atrial fibrillation (AF) and resultant thromboembolism. Even patients who remain in sinus rhythm appear to have an increased risk of atrial thrombosis and thromboembolic events in the context of CA.^3^

Diagnosing amyloidosis can be challenging due to its broad and often nonspecific clinical presentation. A high index of suspicion is therefore necessary to prompt further diagnostic evaluation.^3^ Case reports in the literature have described delays of up to 12 months from initial suspicion of amyloidosis to confirmed diagnosis.^4^ The definitive diagnosis of amyloidosis requires histologic confirmation from a biopsy specimen of any involved tissue, demonstrating Congo red positivity with apple-green birefringence under polarized light.^5^ However, ATTR cardiomyopathy (ATTR-CM) can be diagnosed non-invasively with a bone scintigraphy once a monoclonal gammopathy has been excluded. Planar technetium-99m pyrophosphate (PYP) is the primary imaging modality used. Increased cardiac uptake of the radioactive tracer, either by visual grading or by measuring a planar 1-h heart-to-contralateral lung (H/CL) ratio of >1.4 or Grade 2, has been shown to exhibit high sensitivity and specificity for diagnosing ATTR.^6^

In this case series, we present three patients in whom the diagnosis of amyloidosis carries a critical learning opportunity. These cases highlight the variability of clinical presentations, limitations of standard diagnostic approaches, and potential for delayed diagnosis due to subtle clinical suspicion.

## Summary figure

**Figure:**
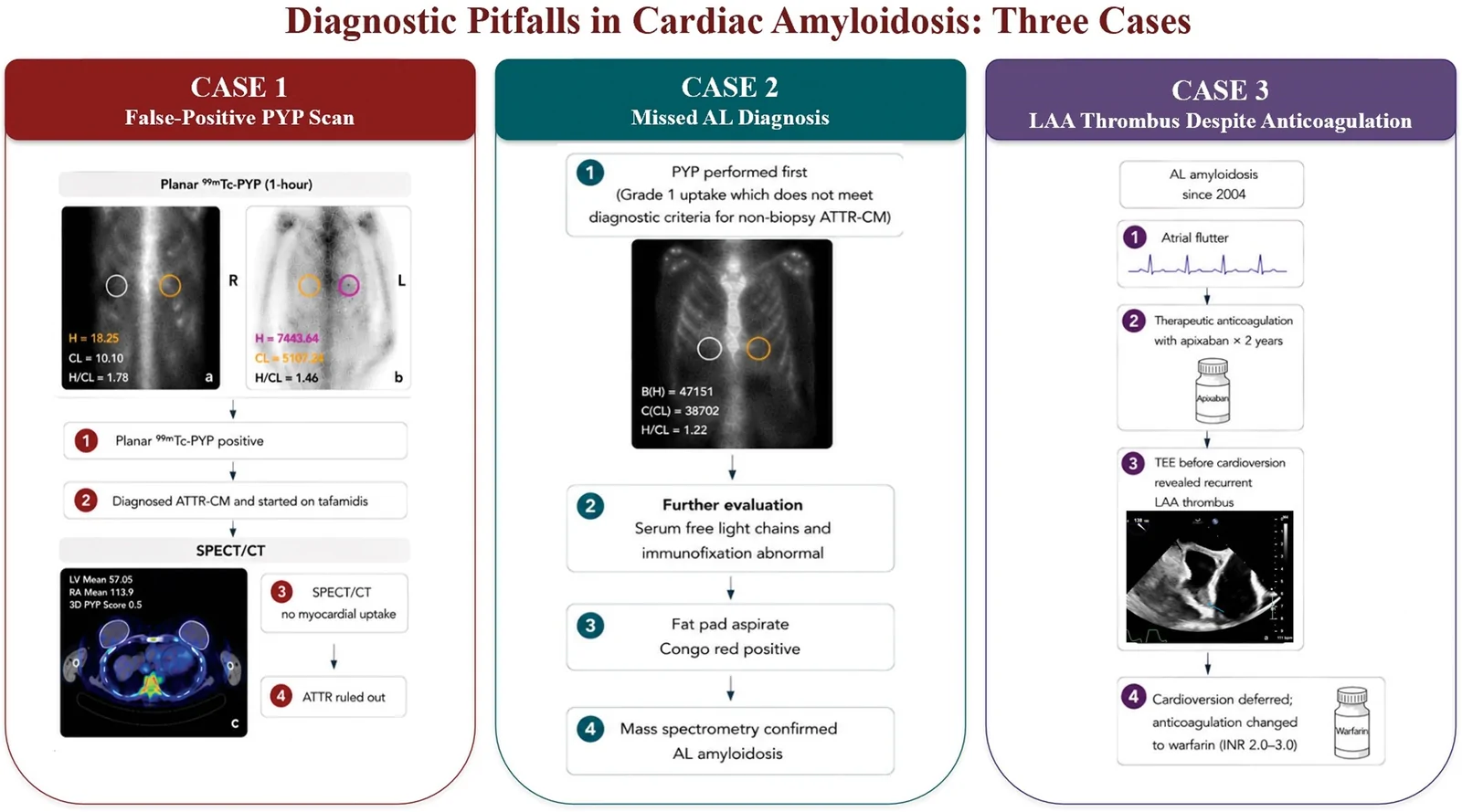


## Case 1

### History of presentation

**Figure 1:**
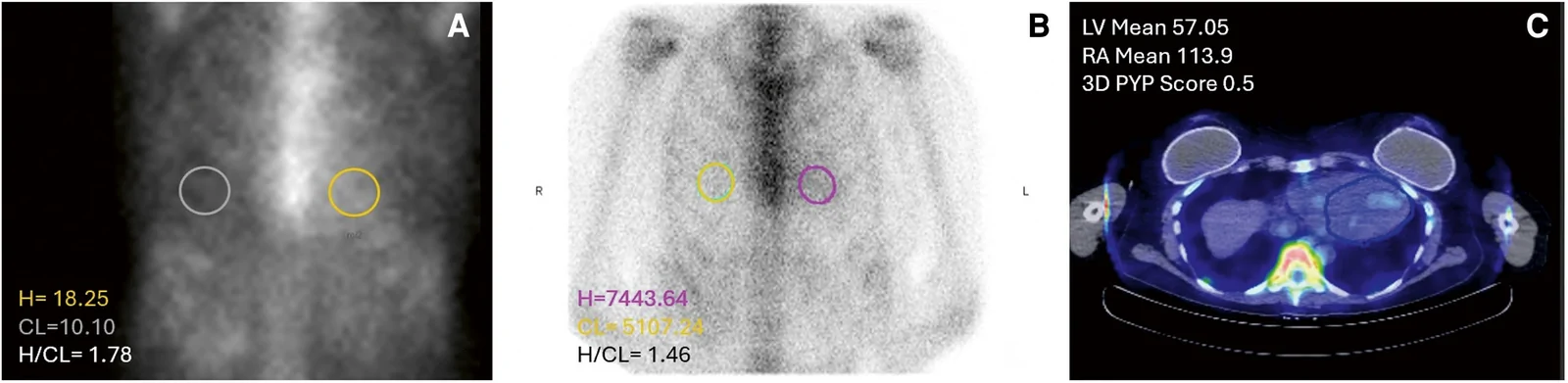
PYP scintigraphy. (*A*) Planar 1-h imaging at an outside facility showed an H/CL ratio of 1.78 (positive ≥ 1.5). (*B*) Repeat planar imaging at 3 h demonstrated an H/CL ratio of 1.46 (positive > 1.3). (*C*) Corresponding SPECT/CT revealed no myocardial uptake, with a 3D PYP score of 0.5 (positive>1.1), confirming the absence of cardiac amyloid uptake. SPECT, single-photon emission computed tomography; PYP, planar technetium-99m pyrophosphate; H/CL, heart-to-contralateral lung.

A 67-year-old woman initially presented in 2019 with progressively worsening dyspnoea on exertion. Transthoracic echocardiography (TTE) was performed and demonstrated a reduced left ventricular ejection fraction (LVEF) of 25%, regional wall motion abnormalities characterized by akinesis of the anterior wall with relatively preserved contractility of the inferior wall, ventricular septal dyssynchrony, and normal left ventricular wall thickness. Electrocardiography (ECG) showed sinus bradycardia with criteria for left ventricular (LV) hypertrophy and secondary ST-T abnormalities. Given the presence of regional wall motion abnormalities and reduced LVEF, coronary angiography was performed and revealed no obstructive coronary artery disease. Further evaluation with cardiac magnetic resonance was planned; however, despite multiple attempts and the use of sedation, the patient was unable to tolerate the examination because of severe claustrophobia. Consequently, a PYP scan was performed, which demonstrated an H/CL ratio of 1.78 (positive ≥ 1.5) (*Figure 1A*). This H/CL ratio provides a semi-quantitative measure of myocardial amyloid deposition. An H/CL ratio of ≥1.5 at 1 h (or ≥1.3 at 3 h) is highly suggestive of the diagnosis of ATTR-CM. Monoclonal component screening, including serum free light chain analysis and serum/urine immunofixation studies, was not obtained at the first institution. She was subsequently diagnosed with ATTR-CM and started on tafamidis; an oral selective transthyretin stabilizer indicated to slow disease progression.

### Past medical history

Her medical comorbidities include dyslipidaemia, Type 2 diabetes mellitus, obesity, and chronic kidney disease Stage III. Her surgical history included bilateral silicone breast implantation.

### Differential diagnosis

The differential diagnosis included ATTR-CM and non-ischaemic cardiomyopathy secondary to long-standing hypertension.

### Investigations

For a second opinion, she presented to another institution. Initial workup with a TTE was performed and demonstrated a LVEF of 27% and mild LV dilatation, with an end-diastolic diameter of 58 mm. The interventricular septum and posterior wall measured 9 mm (normal range: 6–10 mm), indicating the absence of increased wall thickness. Global longitudinal strain was reduced, averaging −9% (normal = more negative than −18%), with relative apical sparing, a pattern that can be seen in CA. A PYP scan was performed and at 3 h, the H/CL ratio on anterior planar imaging was 1.46 (positive > 1.3) (*Figure 1B*); however, single-photon emission computed tomography (SPECT) revealed no myocardial uptake, with a 3D PYP score of 0.5 (positive > 1.1) (*Figure 1C*). Abdominal fat pad aspirate was performed and was negative for Congo red staining. Evaluation for amyloid light-chain CA was unrevealing, with a normal serum free κ/λ ratio of 1.13 (normal range: 0.26–1.65), and no monoclonal proteins were detected on serum or urine studies. Cardiac magnetic resonance showed a normal LV size with a reduced LVEF of 38%, no late gadolinium enhancement, and no evidence of non-compaction or myocardial iron overload, with a normal T2 value of >20 ms. Right ventricular size and function were preserved. Endomyocardial biopsy of the right ventricle revealed moderate cardiomyocyte hypertrophy without interstitial fibrosis, amyloidosis, or iron deposition. Lastly, genetic testing identified a heterozygous GAA c.271G>A (p.Asp91Asn) variant, which is classified as a benign pseudodeficiency allele.

### Management

She was diagnosed with idiopathic non-ischaemic cardiomyopathy, likely secondary to longstanding untreated hypertension, and initiated on guideline-directed medical therapy (GDMT).

### Outcomes and follow-up

**Figure 2:**
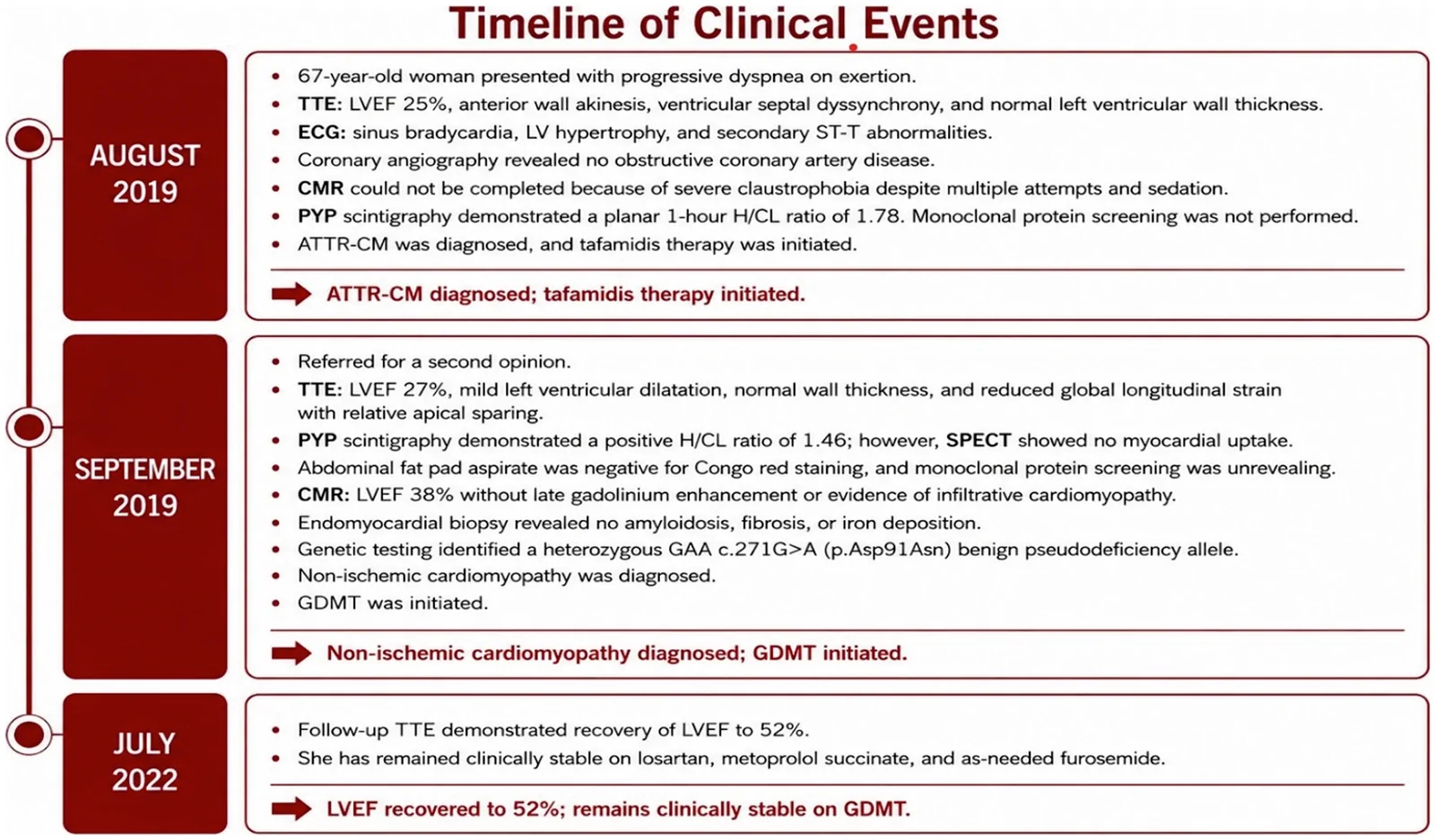
Timeline of clinical events for Case 1.

At 3-year follow-up, her LVEF normalized, and she remained clinically stable on losartan 50 mg twice daily, metoprolol succinate 37.5 mg daily, and furosemide as needed. Refer to *Figure 2* for a timeline of clinical events.

### Take-home messages

- This case highlights the potential for false-positive 2D planar PYP and underscores the importance of confirmatory 3D SPECT before establishing a diagnosis of ATTR-CM and initiating disease-specific therapy.
- The use of 3D SPECT allows for more accurate localization and facilitates accurate differentiation between true myocardial uptake and activity within the cardiac blood pool.
- The false-positive 2D planar PYP finding may be related to silicone breast implants, though this remains speculative.

## Case 2

### History of present illness

**Figure 3:**
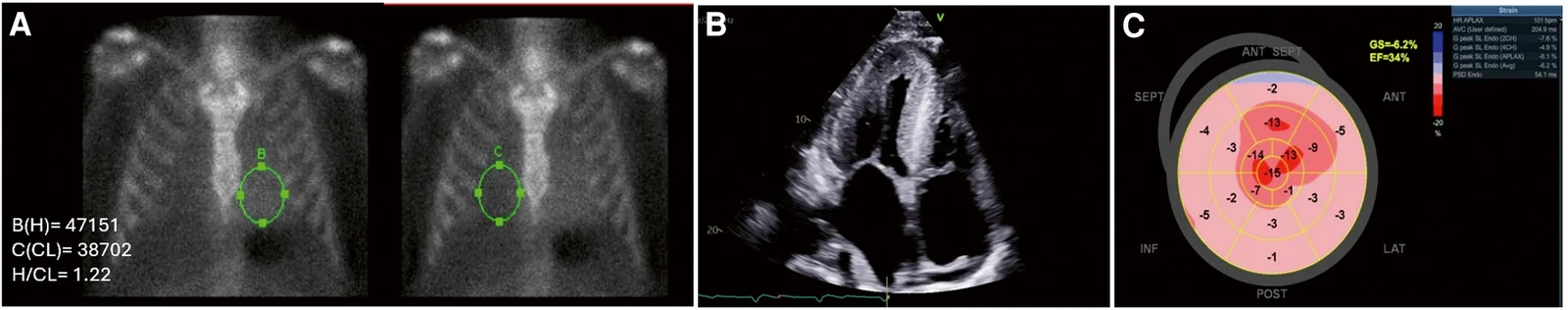
Multimodality cardiac imaging. (*A*) One-hour PYP scan demonstrating an H/CL ratio of 1.22 (negative < 1.5 at 1 h). (*B*) TTE, apical four-chamber view, demonstrating concentric left ventricular wall thickening, biatrial enlargement, thickened interatrial septum, and a granular ‘sparkling’ myocardial appearance suggestive of amyloidosis. (*C*) GLS bull’s-eye plot showing markedly reduced GLS (−6.2%) with relative apical sparing, a characteristic strain pattern in cardiac amyloidosis. PYP, planar technetium-99m pyrophosphate; TTE, transthoracic echocardiogram; GLS, global longitudinal strain; H/CL, heart-to-contralateral lung.

A 69-year-old man presented to an outside facility with lower extremity oedema, initially attributed to venous insufficiency. The patient had a TTE, which revealed increased LV wall thickness with a LVEF of 45%. Electrocardiography demonstrated low-voltage QRS complexes in the limb leads with left axis deviation. He subsequently underwent coronary angiography and left ventriculography, which revealed non-obstructive coronary artery disease, preserved LVEF of 55%–60%, and elevated LV filling pressures. Given the increased LV wall thickness, low-voltage QRS complexes on ECG, unremarkable coronary angiography, and progressive heart failure symptoms, further evaluation for CA was pursued. A PYP scan was performed and demonstrated Grade 1 myocardial uptake, which did not meet criteria for a non-biopsy diagnosis of ATTR-CM (*Figure 3A*). However, no further evaluation with dysproteinaemia testing was performed. He was diagnosed with congestive heart failure due to idiopathic non-ischaemic cardiomyopathy and started on GDMT, including metoprolol succinate 12.5 mg, spironolactone 12.5 mg, and furosemide 40 mg. However, despite therapy, he continued to have dyspnoea on exertion, limiting his ability to walk for more than a block and significant orthopnoea.

### Past medical history

His medical comorbidities include hypertension, dyslipidaemia, history of significant alcohol use disorder, Class I obesity, and obstructive sleep apnoea.

### Investigations

He was referred to a tertiary centre for a second opinion, where he presented with New York Heart Association Class III symptoms, including progressive dyspnoea on exertion, orthopnoea, and bilateral lower extremity oedema. He also endorsed dizziness and headaches, particularly upon standing from a seated position. Orthostatic vital signs revealed a >20 mmHg decrease in systolic blood pressure with standing, consistent with orthostatic hypotension. Initial evaluation with TTE demonstrated a reduced ejection fraction of 43%, increased concentric LV wall thickness, severely increased right ventricular wall thickness measuring 12 mm (normal: 3–4 mm), and severely reduced global averaged LV longitudinal peak systolic strain of −6% (normal: more negative than −18%) (*Figure 3B* and *C*). Subsequent workup with dysproteinaemia testing demonstrated a normal serum kappa free light chain level of 1.39 mg/dL (reference range, 0.33–1.94 mg/dL), a markedly elevated lambda free light-chain level of 143 mg/dL (reference range, 0.57–2.63 mg/dL), and a decreased kappa-to-lambda ratio of 0.0097 (normal range: 0.26–1.65). A serum quantitative M-protein assay demonstrated elevated levels of IgA lambda, while a 24-h urine monoclonal protein study identified increased total protein and lambda monoclonal protein.

Abdominal fat pad aspiration confirmed amyloid deposition by Congo red staining. Liquid chromatography–tandem mass spectrometry performed on peptides extracted from the aspirate identified a peptide profile consistent with AL-type amyloid, confirming the diagnosis of AL amyloidosis. He was referred to haematology/oncology for further evaluation. Bone marrow biopsy with flow cytometry identified a clonal (neoplastic) plasma cell population with an aberrant immunophenotype distinct from normal plasma cells, confirming an underlying plasma cell disorder. Morphology showed 5%–10% lambda light-chain–restricted plasma cells in an otherwise normocellular marrow with preserved trilineage haematopoiesis.

### Outcomes and follow-up

He was initiated on plasma cell-directed therapy with daratumumab and CyBorD, along with dexamethasone 20 mg weekly. Supportive care included acyclovir, Bactrim, and levofloxacin prophylaxis. The treatment goal was to achieve a rapid and very good partial response, reflected by a reduction in lambda light chains.

**Figure 4:**
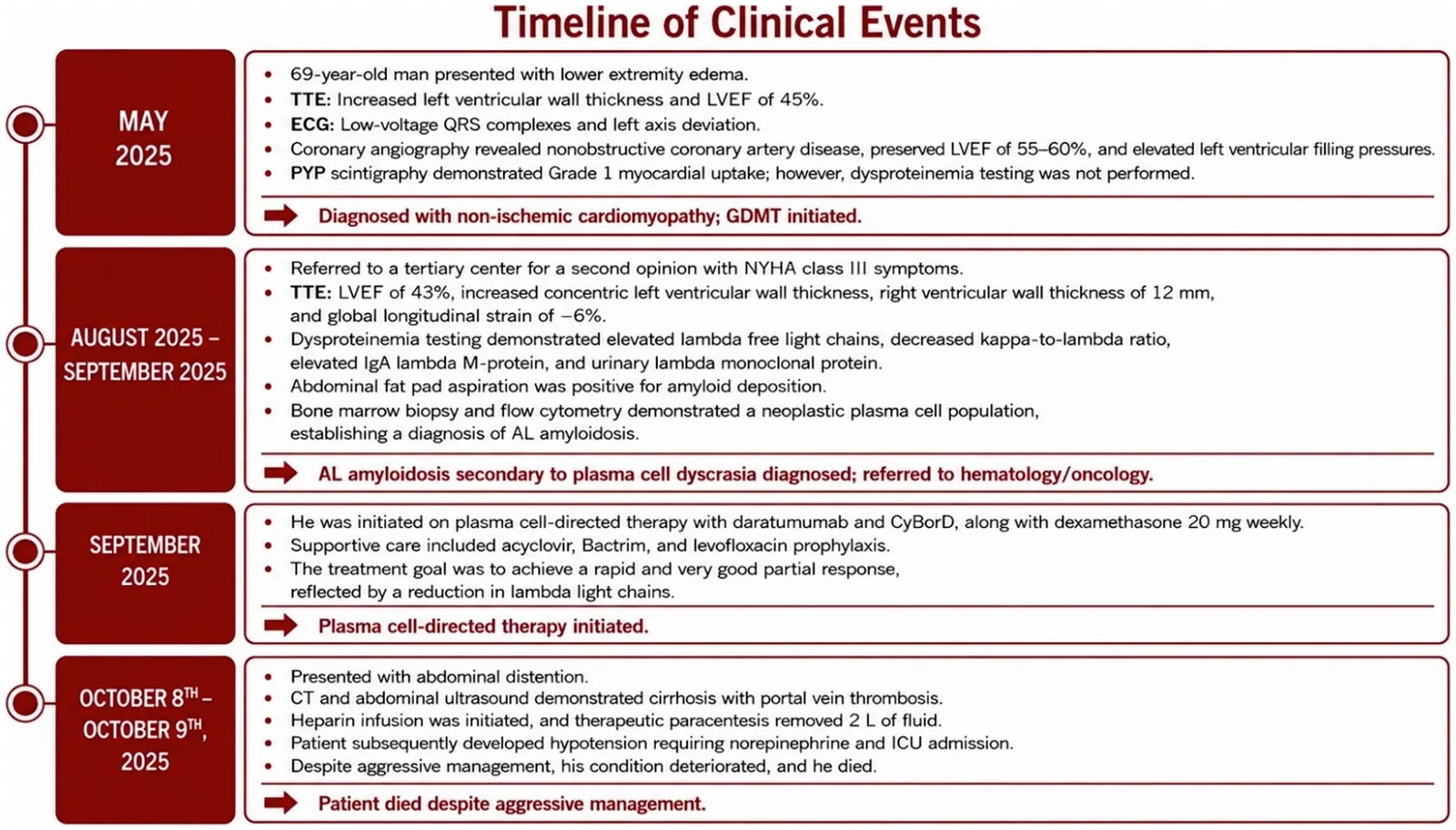
Timeline of clinical events for Case 2.

Approximately 1 month later, the patient presented to the emergency department with abdominal distention. Computed tomography imaging revealed cirrhotic changes in the liver and evidence of portal vein thrombosis, which was subsequently confirmed by abdominal ultrasound. A heparin infusion was initiated, and a therapeutic paracentesis was performed, removing 2 L of fluid. Following the procedure, the patient developed hypotension requiring vasopressor support with norepinephrine and was transferred to the intensive care unit. Despite aggressive management, the patient’s condition deteriorated, and he died. Refer to *Figure 4* for a timeline of clinical events.

### Take-home messages

- Comprehensive evaluation for CA requires assessment for both ATTR and AL types: PYP scintigraphy for ATTR and serum and urine monoclonal protein studies with free light chains for AL. Failure to perform both can result in misdiagnosis and delayed initiation of life-prolonging chemotherapy in AL amyloidosis.

## Case 3

### History of present illness

A 54-year-old woman with a history of AL amyloidosis, diagnosed in 2004, presented to the Amyloidosis Clinic for follow-up. She reported a fluttering sensation in her chest, similar to prior episodes of AF experienced in 2023. She also endorsed increased shortness of breath, a dry cough, light-headedness, and worsening bilateral lower extremity oedema but denied chest pain, orthopnoea, and paroxysmal nocturnal dyspnoea.

Two months earlier at a follow-up visit with her primary care provider, she was found to be in AF with a rapid ventricular response (heart rate 137 b.p.m.) and hypotension (blood pressure 79/57 mmHg). She was initiated on metoprolol 25 mg twice daily.

### Past medical history

The patient has a history of systemic AL amyloidosis with multisystem involvement. She was previously treated with melphalan followed by autologous stem cell transplantation, CyBorD, and daratumumab, achieving sustained remission. Her arrhythmic history included AF/flutter, supraventricular tachycardia status post two ablations, and transoesophageal echocardiogram (TEE)-guided direct current cardioversions (DCCV) in 2020 and 2023. A left atrial appendage (LAA) thrombus was identified in 2020, and since then, she remained on long-term anticoagulation, transitioning from warfarin to apixaban in 2023.

### Investigations

Laboratory evaluation demonstrated a significant rise in N-terminal pro-B-type natriuretic peptide to 17 553 pg/mL from 6818 pg/mL ∼1 year prior, consistent with worsening heart failure. Troponin levels were mildly elevated at 66 ng/L compared to 51 ng/L a year ago. Electrocardiography revealed atrial flutter with variable block, with ventricular rates ranging from 100 to 110 b.p.m..

### Management

The patient was started on an amiodarone loading regimen for 2 weeks with plans to proceed with TEE-guided cardioversion. Because of her previous history of LAA thrombus, the decision was made to perform a TEE prior to cardioversion to exclude recurrent thrombus. Metoprolol 25 mg was discontinued, and she was initiated on a loading dose of amiodarone.

### Outcomes and follow-up

**Figure 5:**
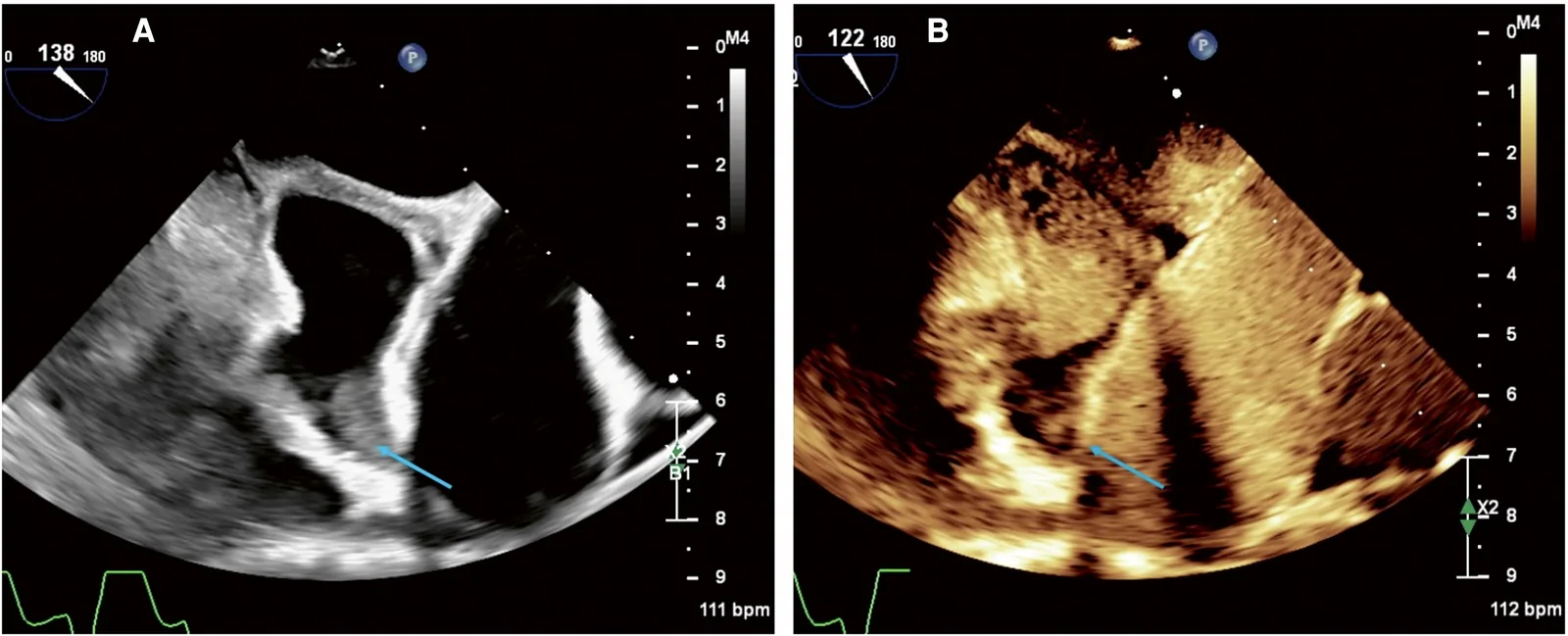
TEE images prior to DCCV. (*A*) Mid-oesophageal view demonstrating a filling defect within the LAA, suspicious for thrombus (arrow). (*B*) Contrast-enhanced TEE confirming the presence of thrombus with lack of contrast opacification in the appendage (arrow). TEE, transoesophageal echocardiogram; LAA, left atrial appendage; DCCV, direct current cardioversions.

**Figure 6:**
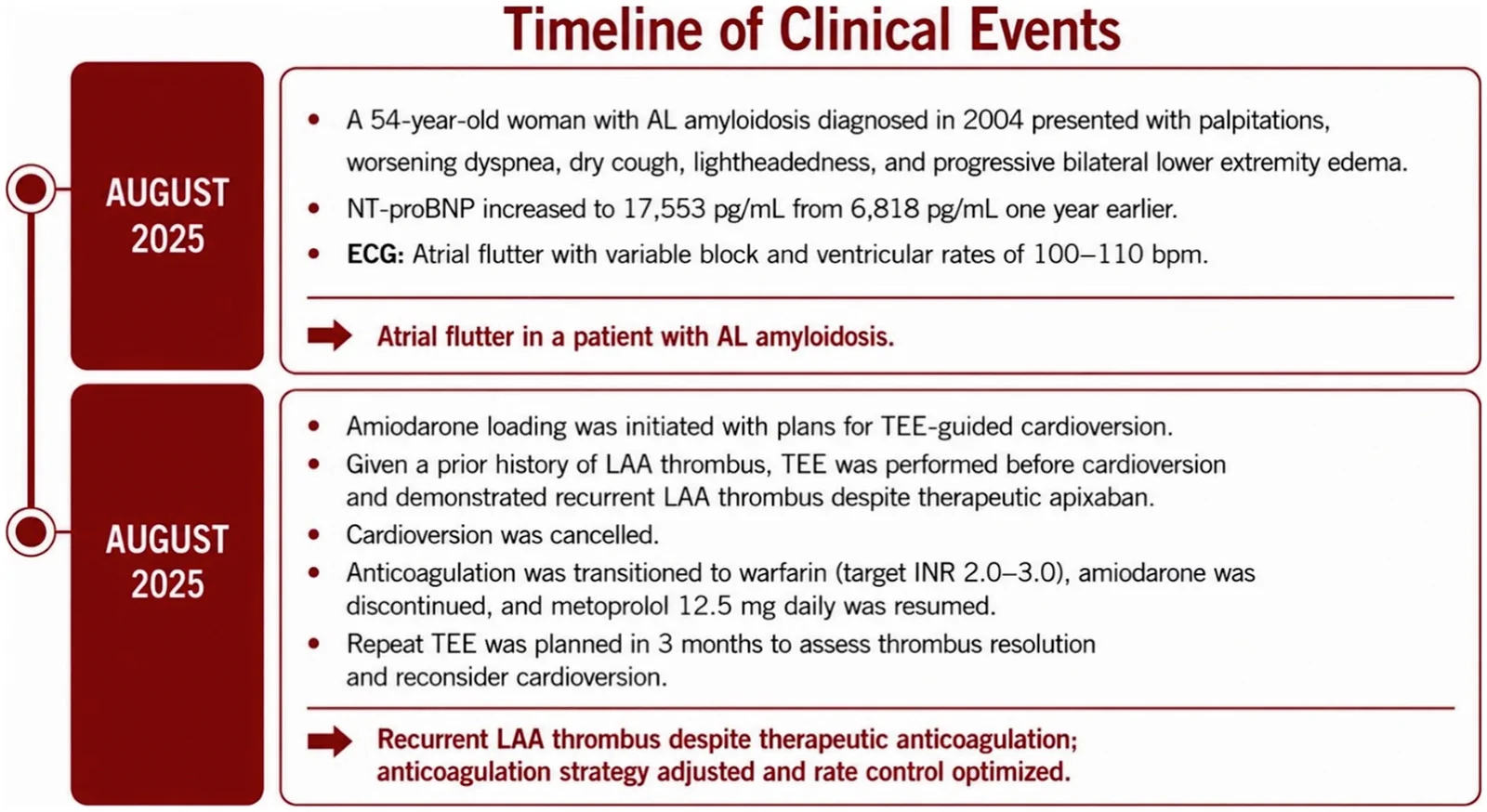
Timeline of clinical events for Case 3.

Despite being on therapeutic anticoagulation with apixaban for the last 2 years, TEE revealed a thrombus in the LAA (*Figure 5*), prompting the cancellation of planned cardioversion. Given that the thrombus occurred while on apixaban, anticoagulation therapy was transitioned to warfarin, with a target international normalized ratio range of 2.0–3.0. Amiodarone was discontinued, and metoprolol was resumed at a daily dose of 12.5 mg. A repeat TEE was scheduled in 3 months to assess thrombus resolution and reconsider the need for cardioversion. Refer to *Figure 6* for a timeline of clinical events.

### Take-home messages

- This case highlights that in amyloidosis, the atrial substrate is thrombogenic, and TEE should always precede DCCV, even in the setting of therapeutic anticoagulation.

## Discussion

Diagnosing amyloidosis generally requires histological confirmation; however, as previously mentioned, ATTR-CM can be diagnosed non-invasively with a bone scintigraphy once a monoclonal gammopathy has been excluded. False-positive PYP scans are very rare.^7^ Reported instances in the literature include cases associated with hydroxychloroquine use, two patients with hypertrophic cardiomyopathy, and a misleading scan suggestive of ATTR-CM in the setting of acute coronary syndrome.^6–8^ Our first case highlights how a false-positive PYP scan can occur with 2D planar imaging, underscoring the importance of confirmatory 3D SPECT, which has a better ability to accurately localize true myocardial uptake. The most common cause of false-positive planar PYP scans is misinterpretation of blood pool activity as myocardial tracer uptake, particularly in patients with reduced cardiac output.^9^ Single-photon emission computed tomography imaging mitigates this limitation through 3D tomographic assessment, enabling precise localization of radiotracer uptake within the LV myocardium and reliable distinction from blood pool activity.^10^ Furthermore, SPECT can identify other causes of false-positive planar findings, including overlying rib uptake or rib fractures, which may elevate radiotracer counts, alter H/CL ratio calculations, or mimic myocardial uptake.^9^ Acute or subacute myocardial infarction may also produce focal tracer uptake; therefore, scintigraphy should not be used to establish a diagnosis of ATTR-CM in this setting.^9,10^

In addition, the presence of bilateral breast implants can be a contributing factor. Prior literature has reported an increased frequency of abnormal findings on cardiac testing among women with breast implants.^11^ Although this association has not been established, further studies could help determine whether breast implants can interfere with 2D PYP imaging.

Furthermore, delays in diagnosis remain a major challenge, particularly in AL cardiomyopathy; in fact, 20% of patients are not accurately diagnosed until 2 years or more after the onset of symptoms. Similarly, 42% of patients with ATTRwt cardiomyopathy experience a diagnostic delay of over 4 years.^12^ Immunoglobulin light-chain amyloidosis progresses rapidly, necessitating prompt diagnosis and immediate treatment. Approximately 30% of patients with advanced CA die within the first year of diagnosis.^2^ This was evident in our second case, where late-stage AL amyloidosis led to a complicated course and the patient died <2 months after diagnosis.

Lastly, CA is associated with arrhythmias, such as AF, which carry an increased risk of thromboembolism. Patients with ATTR amyloidosis appear to have a higher incidence of both AF and thromboembolism compared to those with AL amyloidosis, partly due to restrictive atrial dysfunction resulting from amyloid infiltration of the atrial wall.^13,14^ This infiltration impairs atrial reservoir function and contractility, predisposing to blood stasis and thrombus formation. The development of AF further exacerbates this risk, emphasizing the importance of anticoagulation therapy.^13^ However, even with adequate anticoagulation, intracardiac thrombosis remains a recognized complication, often necessitating imaging-guided assessment with TEE as highlighted in our third case.

## Conclusion

Amyloidosis exhibits a wide spectrum of clinical presentations that often mimic other conditions, contributing to diagnostic delays or missed diagnoses. Immunoglobulin light-chain amyloidosis progresses rapidly and requires prompt diagnosis and treatment. Although ATTR-CM can be diagnosed non-invasively using PYP scintigraphy, false-positive results may occur, highlighting the importance of confirmatory 3D SPECT imaging. Given the high risk of arrhythmias and thromboembolic events, especially in ATTR, imaging-guided interventions are essential for optimizing patient outcomes.

## Lead author biography


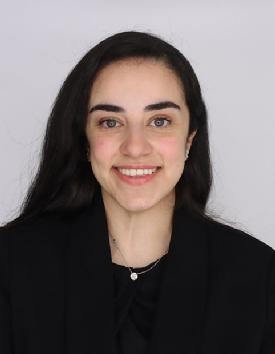


I graduated from the University of Jordan Faculty of Medicine in 2023. Following medical school, I completed 2 years of cardiovascular research at the Mayo Clinic, where I gained extensive experience in both clinical and translational cardiology research. I am currently an internal medicine resident with a strong interest in pursuing a fellowship in cardiology. My career goal is to combine patient-centred clinical care with academic research to advance the prevention and treatment of cardiovascular disease.

## Data Availability

The data underlying this article will be shared on reasonable request to the corresponding author.
